# Development of Eco-Friendly Packaging Films from Soyhull Lignocellulose: Towards Valorizing Agro-Industrial Byproducts

**DOI:** 10.3390/foods13244000

**Published:** 2024-12-11

**Authors:** Sumi Regmi, Sandeep Paudel, Srinivas Janaswamy

**Affiliations:** Department of Dairy and Food Science, South Dakota State University, Brookings, SD 57007, USA; sumi.regmi@jacks.sdstate.edu (S.R.); sandeep.paudel@jacks.sdstate.edu (S.P.)

**Keywords:** agri-industrial byproduct, valorizing soyhulls, lignocellulose extract, sustainable and biodegradable packaging, circular economy

## Abstract

Due to their inability to biodegrade, petroleum-based plastics pose significant environmental challenges by disrupting aquatic, marine, and terrestrial ecosystems. Additionally, the widespread presence of microplastics and nanoplastics induces serious health risks for humans and animals. These pressing issues create an urgent need for designing and developing eco-friendly, biodegradable, renewable, and non-toxic plastic alternatives. To this end, agro-industrial byproducts such as soyhulls, which contain 29–50% lignocellulosic residue, are handy. This study extracted lignocellulosic residue from soyhulls using alkali treatment, dissolved it in ZnCl_2_ solution, and crosslinked it with calcium ions and glycerol to create biodegradable films. The film formulation was optimized using the Box–Behnken design, with response to tensile strength (TS), elongation at break (EB), and water vapor permeability (WVP). The optimized films were further characterized for color, light transmittance, UV-blocking capacity, water absorption, contact angle, and biodegradability. The resulting optimized film demonstrated a tensile strength of 10.4 ± 1.0 MPa, an elongation at break of 9.4 ± 1.8%, and a WVP of 3.5 ± 0.4 × 10^−11^ g·m^−1^·s^−1^·Pa^−1^. Importantly, 90% of the film degrades within 37 days at 24% soil moisture. This outcome underscores the potential of soyhull-derived films as a sustainable, innovative alternative to plastic packaging, contributing to the circular economy and generating additional income for farmers and allied industries.

## 1. Introduction

Fossil-based plastic is an excellent material for packaging applications. Its high strength, low cost, lightweight, and versatility have rapidly increased global demand and production exceeding 400 million tons annually (statista.com). It is an effective thermal insulator, enhancing building energy efficiency, and lightweight plastics in vehicles and airplanes are crucial for increasing fuel efficiency [1]. Its packaging helps prolong the freshness of perishable foods and minimizes food waste. Due to its widespread applications, life without plastic is unimaginable. However, only 9% of the total plastic produced is recycled and 12% is incinerated, and the remaining 79% end up in landfills [2]. Ironically, plastic waste from food packaging accumulates much faster than in other sectors like construction as food packaging has a very short lifespan, often limited to the duration of food consumption [3,4,5].

Plastic’s unique chemical and physical properties make it difficult to degrade naturally and persist in landfills for hundreds of years. It constitutes most of the marine pollution, making up over 85% of all waste found in the ocean. Every year, around 14 million metric tons of plastic waste are dumped into the seas, and this figure is projected to triple within the next two decades (genevaenvironmentnetwork.org). It fragments into tiny particles of microplastics and nanoplastics. Due to their small size, these particles can accumulate in organisms. In aquatic and wildlife, they may cause harm by triggering inflammation, oxidative stress, and hormonal disruptions. They often carry harmful additives like softeners and flame retardants, which can further induce adverse health effects, such as cardiovascular problems, cancer, metabolic disorders, and reproductive health concerns [6,7,8,9,10,11,12]. These smaller particles can also attract toxic substances from water, increasing the safety risk to marine life.

Several alternate food packaging systems that are biodegradable, non-toxic, and sustainable are being explored worldwide [13]. A few film examples include sweet potato starch [14], rice starch [15], cassava starch [16], oat starch [17], sunflower protein isolate [18], fish myofibrillar protein [19], soybean protein isolate [20], and gelatin [21]. However, considering the continuously growing global population, using human food resources, starch, and protein to develop plastic alternatives is not rational, prudent, or sustainable. In this regard, an eco-friendlier approach that uses alternative, non-food-based sources like lignocellulose from biomass and agro-industrial byproducts is prudent [22,23,24,25,26]. To this end, renewable agro-processing byproducts such as soyhulls can be feasible materials for designing and developing biodegradable packaging films.

Soybeans are one of the most valuable oilseed crops. The soybean seeds consist of 7–8% soyhulls, representing the most significant byproduct in the processing industry. Soyhulls incorporated in livestock feed are considered high-quality feed for cattle and have gained widespread use [27]. Additionally, they serve as effective binding materials for heavy metals in wastewater treatment [28]. Incorporating hulls as dietary fiber controls obesity and promotes general health. Furthermore, the hulls have applications in producing ethanol and bio-oil [29,30]. However, 29–51% of cellulosic residue in soyhulls offers an undeniable opportunity to address plastic concerns [22,31]. The worldwide production of soybeans will reach approximately 411 million tons by 2030 [32]. Thus, a significant amount of cellulosic material could be obtained for creating sustainable and functional products. The global production of soybeans in 2023/24 was 398.2 million metric tons, among which the US produced 113 million metric tons (statista.com). In the US, soybeans are the second most produced crop after corn [33]. Only from the US production, it translates to roughly 7.9–9.1 million tons of hulls. This generates a valuable source of 2.4–4.6 million tons of cellulosic residue. The livestock feed value of soyhulls is around USD 170–225 per ton [34]. Several soybean-crushing plants also add hulls back to soymeal; consequently, the hull value could increase. More value addition could be envisioned by developing biodegradable plastic films and products made from the cellulosic residue from the soyhulls. This helps to address current and future plastic concerns and improve environmental and human health.

This study aimed to extract lignocellulosic residue from soyhulls (SHE) using a 50% NaOH solution to develop, optimize, and characterize biodegradable films. The outcome of this research strategy promotes a waste-free solution by introducing innovative biodegradable packaging options, conserving the environment while creating new income paths for farmers and industries.

## 2. Materials and Methods

### 2.1. Materials

Soyhull was a gift from the South Dakota Soybean Processors, Volga, South Dakota, USA. Ethanol was obtained from the Department of Chemistry and Biochemistry at South Dakota State University. The chemicals calcium sulfate, calcium chloride dihydrate, sodium hydroxide, potassium sulfate, glycerol, and zinc chloride were bought from VWR International, Radnor, PA, USA.

### 2.2. Methods

#### 2.2.1. Extraction of Lignocellulosic Residue

The extraction of lignocellulosic residue, film preparation, and optimization followed the published protocol [35], with modifications. Briefly, soyhulls were ground to a 60-mesh size using a Glen Mill grinder. The powder of 125 g was treated with 1600 mL of 50% NaOH solution (*w*/*v*) and stirred at 320 rpm at 35 °C for 8 h. The mixture was then filtered through a 100-mesh sieve and washed with distilled water until a neutral pH was achieved. The resulting soyhull lignocellulosic residue extract (SHE) was dried at 40 °C for 12 h in a hot air oven. The dried residue yield was 33.3 ± 1.7%. It was ground to a 60-mesh size using a Lafati 300A high-speed multi-functional crusher and stored at room temperature (RT, 22 ± 2 °C) in an airtight jar for further use.

#### 2.2.2. Film Preparation

The SHE was used for film preparation following the published protocol and Box–Behnken Design (BBD) [36,37]. To prepare the film solution, 0.3–0.5 g of SHE was swelled in a glass vial with 1.6 mL of distilled water in a water bath (90 °C) for 2 h. It was then solubilized in a 68% ZnCl_2_ solution for 30 min at 83 °C, crosslinked with 200–500 mM CaCl_2_ for 10 min, and plasticized with 0.5–1.5% glycerol for 5 min. Preliminary experiments were conducted to determine the suitable SHE, CaCl_2_, and glycerol range for BBD optimization. The resulting solution was manually cast onto a 10 × 12-inch glass plate placed in a tray using a handheld applicator with a 1 mm clearance. Film regeneration was achieved by adding 500 mL of ethanol to the tray and shaking it at 50 rpm for 5 min on a VWR advanced digital shaker (model 3500, 89032-096, VWR International, USA). Later, the film was framed using wooden strips, immersed in distilled water for 10 min, dried at RT for 24 h, and stored in airtight Ziploc bags at RT for further characterization.

#### 2.2.3. Film Optimization

In this study, SHE, CaCl_2_, and glycerol were the independent variables for the BBD, which included 15 experimental combinations. The responses used to optimize the film formulation were tensile strength (TS), elongation at break (EB), and water vapor permeability (WVP). Optimization was carried out using the ANOVA-quadratic model by evaluating the responses with model fitness confirmed by a *p*-value (<0.05) and a high coefficient of determination (R^2^). The optimal film formulation was determined by maximizing TS and EB and minimizing WVP.

The TS and EB were measured using a film strip measuring 1×8 cm and secured in the grips of a Texture Analyzer (Stable Micro Systems, Model TA-HD plus, serial no: 5529, Surrey, UK) with a 6 cm gap between the grips. The test was conducted with a trigger force of 50 N and an elongation speed of 15 mm/s. TS and EB were determined using Equations (1) and (2), respectively.

(1) $$TS\;(MPa)=\frac{Force\;used\;to\;break\;film\;(N)}{Film’s\;surface\;area\;({mm}^{2})}$$

(2) $$EB\;(\%)=\frac{Elongation\;at\;break\;(mm)}{Initial\;length\;(mm)}\times 100$$

To determine WVP, a film strip was sealed on a glass vial containing 4 g of anhydrous CaSO_4_, ensuring a relative humidity (RH) of 0%. The vial was placed in a desiccator with a saturated K_2_SO_4_ solution to maintain 97% RH. The experiment was conducted at 25 °C, and the vial’s weight was recorded hourly over an 8 h period. The water vapor transmission rate (WVTR) was determined as the rate of weight change per unit time and the surface area of the film, and WVP was calculated using Equation (3). The thickness of the film was determined with a digital micrometer vernier caliper (RexBeti, Auburn, WA, USA).

(3) $$WVP=\frac{WVTR}{P\;(R_{1}-R_{2})}\times t$$

Herein, P: saturation vapor pressure of water (Pa) at 25 °C; R_1_: RH in the desiccator; R_2_: RH in the cup; t: film thickness (m); and the driving force [$P\;(R_{1}-R_{2})$] is 3073.93 Pa in the experimental condition.

#### 2.2.4. Optimized Film Characterization

The optimized film was prepared and measured for TS, EB, and WVP to test the model’s fitting by comparing them with the predicted values. Then, the film was characterized for color, spectroscopic, hydration properties, and soil biodegradability using the established protocol [35,37,38].

##### Color Properties

The Hunter L* a* b* scale was used for color measurement employing a Nix Pro 2 color sensor (Model no: NIXPRO002, Nix Sensor Ltd., Hamilton, ON, Canada) and a standard white plate as a background. The whiteness index, yellowness index, and total color difference were calculated using Equations (4)–(6).

(4) $$WI={100-[{\left(100-L*\right)}^{2}+{a*}^{2}+{b*}^{2}]}^{0.5}$$

(5) $$YI=142.86\frac{b*}{L*}$$

(6) $$TCD={\left[{\left(L*-{L*}_{b}\right)}^{2}+{\left(a*-{a*}_{b}\right)}^{2}+{\left(b*-{b*}_{b}\right)}^{2}\right]}^{0.5}$$

Herein, L*, a*, and b*: film’s color; L*_b_, a*_b_, and b*_b_: background color; WI: whiteness index; YI: yellowness index; and TCD: total color difference.

##### Spectroscopic Properties

FTIR spectra were collected using a PerkinElmer Spectrum 100 spectrophotometer in the 400 to 4000 cm^−1^ wavenumber range. Herein, 36 scans at 4 cm^−1^ resolution were carried out. The first derivative was used to identify the peak position.

Electromagnetic radiation transmittance was analyzed with a UV-Vis spectrophotometer (Model UV-1600PC, 10037-436, VWR International, USA) in the 190 to 1060 nm wavelength, recorded at 10 nm intervals. A blank cuvette, set to 100% transmittance, was the reference. The transparency and absorption coefficient (α) were determined using Equations (7) and (8).

(7) $$Transparency=\frac{\log\left(T_{600}\right)}{t}$$

(8) $$\alpha =\frac{1}{t}ln\left(\frac{1}{T\%}\right)$$

Herein, T_600_: transmittance at 600 nm (%); t: the thickness of the film (mm); α: absorption coefficient (mm^−1^); and T%: transmittance.

##### Antioxidant Activity

The antioxidant activity of the optimized film was evaluated through a DPPH radical scavenging assay. Approximately 3 g of the film was cut into small pieces and stirred in 100 mL of ethanol at RT, using a magnetic stirrer set to 100 rpm in a glass beaker for one hour. The resulting film–ethanol mixture was serially diluted to prepare solutions with film-to-ethanol ratios of 0.03, 0.015, 0.0075, 0.00375, and 0.0009375 g/mL. Furthermore, 1 mL solution from each dilution was transferred into individual vials, and 3 mL of a 0.1 mM DPPH solution was added to each vial. The solutions were thoroughly vortexed and placed in the dark for 30 min. A blank was prepared using absolute ethanol. The absorbance of each sample was recorded at 517 nm using a UV-Vis spectrophotometer. The percentage of radical scavenging was determined using Equation (9), and the IC50 value, representing the concentration required to achieve 50% radical scavenging, was calculated using a linear regression model.

(9) $$Radical\;Scavenging(\%)=\left(1-\frac{Absorbance\;of\;sample}{Absorbance\;of\;control}\right)\times 100$$

##### Hydration Properties

The moisture content was determined by drying the film in a hot air oven at 110 °C for 24 h and calculating it using Equation (10).

(10) $$Moisture\left(\%\right)=\left(\frac{Initial\;weight-Weight\;after\;drying}{Initial\;weight}\right)\times 100$$

The water solubility of the film was determined by using 2 × 2 cm film strips. They were dried in a hot air oven at 110 °C for 24 h and weighed. Later, the strips were immersed in 50 mL of distilled water and shaken continuously at 175 rpm for 24 h. Subsequently, the strips were dried again, and their final weight was recorded. The percentage of water solubility was calculated using Equation (11).

(11) $$Water\;solubility\left(\%\right)=\frac{\left(Initial\;dried\;weight-Final\;dried\;weight\right)}{Initial\;dried\;weight}\times 100$$

The water absorption behavior of the film was evaluated by immersing a dried 2 × 2 cm film strip in 100 mL of distilled water. The strip was periodically removed at 5, 10, 15, 30, 60, 90, and 120 min, the surface was gently blotted to remove excess water, and the weight was recorded. The percentage of water absorption was calculated using Equation (12). To analyze the water absorption kinetics, nine established models, namely Peleg, Singh, Gornicki, Pilosof, Czel and Czigany, Peppas, Vega-Galvez, Garcia-Pascual, and Weibull (Equations (13)–(21)), were used. Model fitness was assessed using the coefficient of determination (R^2^) and root mean square error (RMSE), calculated through Equations (22) and (23).

(12) $$Water\;absorption\left(\%\right)=\frac{Water\;absorped\;weight-Initial\;dried\;weight}{Initial\;dried\;weight}\times 100$$

(13) $$\frac{t}{{mr}_{t}}=K_{2}t+K_{1}$$

(14) $${mr}_{t}=a+\frac{b\times t}{c\times t+1}$$

(15) $${mr}_{t}=a+b\times (1-\frac{1}{1+b\times c\times t})$$

(16) $${mr}_{t}=a+\frac{b\times t}{c+t}$$

(17) $${mr}_{t}=a\times t^{m}$$

(18) mrtmr∞t12=K1t12+K2

(19) $${mr}_{t}=a\times exp\left[-\frac{b}{{\left(1+t\right)}^{\alpha }}\right]$$

(20) $${mr}_{t}=1-exp\left[{\left(-\frac{t}{a}\right)}^{b}\right]$$

(21) mrt=me×mo−meexp−tβα

(22) $$R^{2}=1-\frac{\sum {{(y}_{i}-\hat{y})}^{2}}{\sum {{(y}_{i}-\bar{y})}^{2}}$$

(23) $$RMSE=\sqrt{\frac{\sum {{(y}_{i}-\hat{y})}^{2}}{N}}$$

Herein, mr_t_: moisture ratio at time ‘t’; mr_∞_ and m_e_: moisture ratio at 120 min; m: power of curve fitting in Czel and Czigany model in power equation; K_1_ and K_2_: intercept and slope of curve fitting in Peleg model, respectively, and slope and intercept of curve fitting in Peppas model, respectively; and a, b, c, α, and β: constants (α = 0.9, a fixed value for the Vega-Galvez’s model only).

The water contact angle was measured using a Dropometer (Droplet Lab, Markham, ON, Canada) with a 0.05 μL precision dropper. A sessile water droplet was placed on the film surface, and its image was captured using a smartphone. The captured image was analyzed with the sessile drop software to calculate the water contact angle. Measurements were also taken at intervals of 10 s, 20 s, and 30 s to evaluate changes in the film’s hydrophobicity as a function of time.

##### Soil Biodegradation

To study biodegradation, the soil’s initial moisture content was measured and adjusted to 24 ± 2% by adding the required amount of water, calculated using the Pearson square method. This moisture amount was maintained throughout the experiment. A 3 × 3 cm film strip was weighed and buried in the soil in a cup. The study was conducted at a 22 ± 2 °C and 47 ± 2% relative humidity for triplicate samples. The film’s weight was recorded every 2nd day, and the percentage of biodegradation was determined using Equation (24). First-order and second-order reaction kinetics were applied using Equations (25) and (26). Model fitness was assessed based on the R^2^ and RMSE values, calculated from Equations (22) and (23), with the best-fitting model used to estimate the film’s half-life.

(24) $$\%Biodegradation=\frac{Loss\;in\;film\;weight\times 100}{Initial\;weight}$$

(25) Ln(y)=mx+c

(26) $$Ln(y)=a+bx+cx^{2}$$

wherein, Ln(y): natural logarithm of %biodegradation; x: biodegradation days; m and c: slope and intercept, respectively, in Equation (25); and a, b, and c: constants in Equation (26).

#### 2.2.5. Statistical Analysis

All parameters in the study were measured in triplicate, and the average values are reported (average value ± standard deviation). Experimental design and model fitting for tensile strength (TS), elongation at break (EB), water vapor permeability (WVP), and film optimization were carried out using Stat-Ease 360 Software (Version: 23.1.4). To compare the actual and predicted responses of the optimized film, a Welch two-sample *t*-test was conducted using RStudio 2022.07.01. Water absorption and soil biodegradation kinetics were analyzed using Microsoft Excel for Mac Version 16.24 with Solver Add-ins.

## 3. Results and Discussion

### 3.1. Optimization of Film Combination

**Figure 1:**
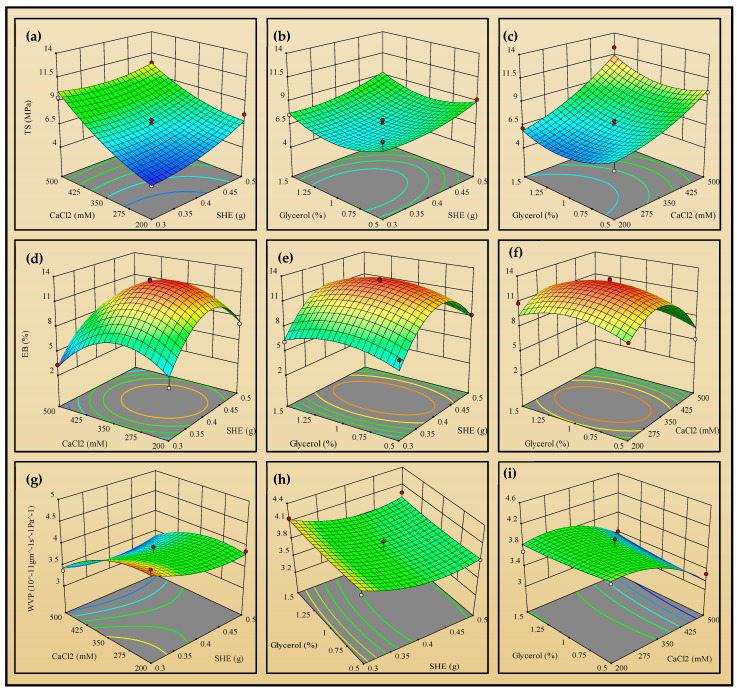
The 3D contour plot showing the linear and interactive effect of the independent variables: soyhull lignocellulosic residue extract (SHE), CaCl_2_, and glycerol, with response to (**a**–**c**) tensile strength (TS), (**d**–**f**) elongation at break (EB), and (**g**–**i**) water vapor permeability (WVP) of the films. The lower to higher values in the graph are represented with blue to red.

The optimization of SHE, CaCl_2_, and glycerol amounts was carried out based on the most important properties of the packaging films: tensile strength (TS), elongation at break (EB), and water vapor permeability (WVP). Table 1 depicts the values of responses of all 15 sample runs. The TS (expressed in MPa) of SHE films ranges from 4.1 ± 0.7 to 12.3 ± 0.1. The variation in SHE, CaCl_2_, and glycerol impacts the TS (Figure 1a–c). The TS of the films increases with a higher SHE content, as observed with a TS of 4.1 ± 0.7 in SHE1 (0.3 g SHE), which rises to 7.6 ± 1.2 MPa in SHE2 (0.5 g SHE). Similarly, CaCl_2_ improves the TS; for example, SHE1 with 200 mM CaCl_2_ has a TS of 4.1 ± 0.7, which increases to 9.4 ± 2.9 in SHE3 with 500 mM CaCl_2_. The improved TS with increasing CaCl_2_ concentrations is due to the strong crosslinking of Zn-cellulosic chains in the presence of Ca^2+^ ions. In addition, with the third independent variable, glycerol, the TS slightly decreases, as seen in SHE5 with 0.5% glycerol; the TS is 8.4 ± 1.4, which drops to 7.5 ± 2.7 in SHE7 with 1.5% glycerol. Furthermore, the linear, interactive, and quadratic effect of SHE, CaCl_2_, and glycerol is illustrated in Figure 1a–c, and Equation (27) was used for optimization. Table 2 presents the ANOVA results for the response surface model. A *p*-value of 0.0236 (<0.05) supports the quadratic model fitting. The model’s high R^2^ value of 0.9251 and the lack of fit *p*-value of 0.1529 (>0.05) indicate a good fit.

TS = 6.52 + 0.77A + 2.34B + 0.15C − 0.61AB + 0.03AC + 0.43BC + 0.60A^2^ + 0.74B^2^ + 1.27C^2^ (27)

Herein, A, B, and C correspond to SHE, CaCl_2_, and glycerol, respectively (in terms of the coded number).

The SHE film demonstrates a TS greater than that of low-density polyethylene (7, ref. [39]), making it a strong material for packaging applications. Its TS aligns with the values reported for films from other biomaterials, such as alginate (0.7–2.1, ref. [40]), cellulose (0.8–22.4, ref. [37]), pectin (14.8–19.0, ref. [41]), spent coffee grounds (8.4–26.8, ref. [42]), soyhulls cellulose (0.7–6.3, ref. [35]), and corncob cellulose (1.1–6.8, ref. [38]). Table 3 lists the comparable TS values of films from additional cellulose-based films.

The elongation at break (EB) of 15 sample runs is 4.2 ± 0.4 to 13.1 ± 0.7%. The EB is also influenced by the independent variables. As highlighted in Table 1, an increase in SHE from 0.3 g in SHE3 to 0.5 g in SHE4 increases the EB from 3.3 ± 1.5% to 5.7 ± 0.7%, respectively. The Ca^2+^ ions slightly decrease the EB. For example, EB is 4.2 ± 0.4% in SHE1 with 200 mM CaCl_2_, which decreases to 3.3 ± 1.5% in SHE3 with 500 mM CaCl_2_. Furthermore, the addition of plasticizer glycerol enhances EB. With the increment of glycerol from 0.5% in SHE10 to 1.5% in SHE12, the EB is enhanced from 5.1 ± 1.5 to 6.4 ± 1.0%. However, its combined effect with SHE and CaCl_2_ further enhances EB. Figure 1d–f illustrates the linear, combined, and quadratic effects of SHE, CaCl_2_, and glycerol. Equation (28) was used for optimization. As shown in Table 2, the *p*-value of 0.0270 (<0.05) further validates the quadratic model fitting with R^2^ of 0.9206. The *p*-value for lack of fit 0.0694 (>0.05) suggests the fitness of the model.

EB = 12.76 + 0.86A − 1.34B + 0.05C − 0.15AB − 0.07AC − 0.07BC − 4.23A^2^ − 3.45B^2^ − 1.44C^2^ (28)

The EB of SHE-optimized film is significantly lower than that of low-density polyethylene (300–900%), but comparable to the films from bagasse cellulose (9.96%) [58], wheat straw fibers (16.4–27.3%) [57], carboxymethylcellulose (17.5–19.4%) [54], microcrystalline cellulose (4.3–13.2%) [37], alkali-digested switchgrass lignocellulose fibers (3.4–4.7%) [56], and switchgrass lignocellulose residue (2.2–2.4%) [55]. Table 3 lists the comparable EB values of films from additional cellulose-based films.

WVP is essential in food packaging as it affects a product’s shelf life, quality, and freshness. Effective moisture control prevents spoilage, maintains texture, and reduces microbial growth. WVP (expressed in the units of ×10^−11^ g·m^−1^·s^−1^·Pa^−1^) is affected by SHE, CaCl_2_, and glycerol. The increase in SHE reduces WVP, thereby improving water barrier property. The increment of SHE from 0.3 g in SHE1 to 0.5 g in SHE2 reduces WVP from 4.4 ± 0.1 to 3.9 ± 0.5, respectively. Similarly, increasing CaCl_2_ lowers WVP; for example, it is 3.9 ± 0.5 in SHE2 with 200 mM CaCl_2_, which drops to 3.3 ± 0.2 in SHE4 with 500 mM CaCl_2_. The Ca^2+^ ions are crosslinkers for lignocellulosic polymers, tightening the structure and decreasing the available space, reducing WVP [35]. Furthermore, glycerol slightly increases WVP from 3.8 ± 0.3 in SHE6 to 4.0 ± 0.1 in SHE8, increasing glycerol from 0.5% to 1.5%. Further 3D contour plots (Figure 1g–i) show the linear, combined, and quadratic effects of SHE, CaCl_2_, and glycerol. Equation (29) was used for optimization, and Table 2 shows the ANOVA for the response model. The *p*-value 0.0374 (<0.05) signifies the quadratic model. The R^2^ 0.9084 and lack of fit *p*-value of 0.4448 (>0.05) further justify model fitness.

WVP = 3.79 − 0.13A − 0.31B + 0.02C + 0.10AB + 0.01AC + 0.08BC + 0.17A^2^ − 0.25B^2^ + 0.03C^2^ (29)

Similar WVP values have been reported in the films from bagasse cellulose of 23 [58], linden mucilage of 22.4–43.9 [59], corncob cellulose of 7–23 [38], and spent coffee grounds lignocellulose of 8.3–18.2 [42]. However, the WVP of low-density polyethylene 6.7 × 10^−14^ g·m^−1^·s^−1^·Pa^−1^ is significantly lower than the biopolymer films due to its nonpolar, hydrophobic nature, and dense molecular structure [39]. Table 3 compares the WVP values of a few cellulose-based films.

The optimization equations determined SHE, CaCl_2_, and glycerol amounts to be 0.41 g, 484.73 mM, and 1.06%, respectively. The predicted values of TS, EB, and WVP are 9.3 MPa, 8.8%, and 3.3 × 10^−11^ g·m^−1^·s^−1^·Pa^−1^, respectively. Subsequently, the optimized film was prepared and tested for TS, EB, and WVP, which yielded the values of 10.4 ± 1.0 MPa, 9.4 ± 1.8%, and 3.5 ± 0.4 × 10^−11^ g·m^−1^·s^−1^·Pa^−1^. These values are compared with the actual values using the Welch Two Sample *t*-test at a 95% confidence level. The *p*-values for the TS, EB, and WVP are 0.3630, 0.7016, and 0.6440, respectively (>0.05), suggesting no statistically significant difference between predicted and actual values. This further validates the model and resulting equations.

### 3.2. Characterization of Optimized Film

#### 3.2.1. Color

The color of packaging material is a powerful tool in influencing consumer perception and purchasing behavior. The color of the optimized film was measured using the Hunter L*, a*, and b* scale as 77.2 ± 1.9, 4.7 ± 1.3, and 10.7 ± 0.6, respectively (Table 4). In the Hunter scale, an “L*” value of more than 50 indicates a white color, a positive “a*” value represents a red color, and a positive “b*” value denotes a yellow color. The L*, a*, and b* values of biodegradable films differ depending on the materials used and the film preparation process. For example, chitosan/polyaniline film exhibited L*, a*, and b* values ranging from 17.29 to 56.16, −19.47 to 5.83, and 0.08 to 28.73, respectively [60].

The WI of the optimized film was 74.4 ± 1.9. A higher WI value indicates a brighter white, which is essential for applications with a clean white appearance. The YI was 19.9 ± 0.9, which measures the tendency of a material to appear yellow. A greater YI value signifies a higher yellowness, which can result from aging, material degradation, or certain additives. The TCD quantifies the difference between two colors in a color space, which is helpful in quality control or color consistency tests. It was calculated to be 17.3 ± 1.6, representing the noticeable color difference in reference to the background color.

#### 3.2.2. FTIR

**Figure 2:**
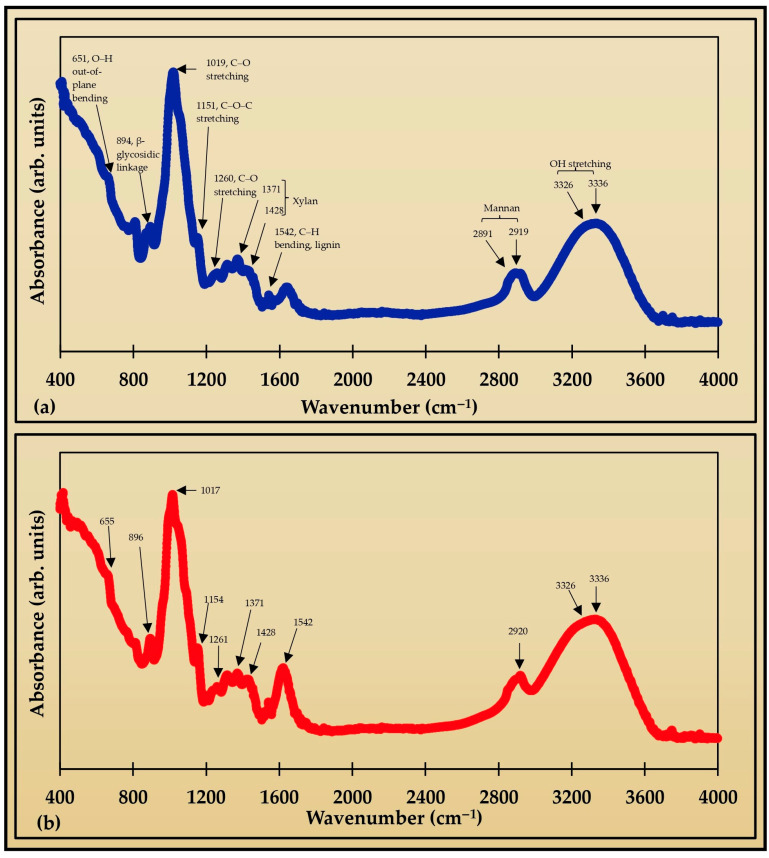
The FTIR peaks of the (**a**) soyhull lignocellulosic extract (SHE) and (**b**) SHE-optimized film. The peaks suggest the presence of cellulose, lignin, mannan, and xylan in the extracted residue, which are also retained in the film.

The significant peaks observed in the optimized SHE film are 655, 813, 896, 1017, 1154, 1261, 1314, 1371, 1428, 1457, 1542, 1623, 2920, 3311, 3326, 3336, and 3750 cm^−1^. The peaks at 1371, 1428, 1457, 1542, 3326, and 3336 cm^−1^ were retained from the soyhulls extract (SHE). Some of the peaks from SHE have shifted and disappeared, and some new peaks formed in the film due to interactions of SHE with ZnCl_2_, CaCl_2_, and glycerol during film preparation (Figure 2). The peaks at 763, 872, 1647, and 2891 cm^−1^ from SHE have disappeared in the film. Additionally, the shifted peaks are 651 to 655, 810 to 813, 894 to 896, 1019 to 1017, 1151 to 1154, 1260 to 1261, 2919 to 2920, and 3310 to 3311 cm^−1^ from SHE to film. The peak 1623 cm^−1^ is the newly formed peak in the film. The 655 cm^−1^ represents O-H out-of-plane bending, and 896 cm^−1^ is present due to the β-glycosidic linkage [61]. Furthermore, the band at 1154 cm^−1^ indicated C-O-C stretching, while 1017 and 1261 correspond to C-O stretching. The 1371 and 1428 cm^−1^ results indicate the presence of xylan, and 1457 and 1542 cm^−1^ represent C-H bending and aromatic ring vibration from lignin. The peak at 2920 cm^−1^ is from mannan with COC, CH_2_, and OH bonds. Similarly, 3326–3336 cm^−1^ are from OH stretching [62,63].

#### 3.2.3. Transmittance, Transparency, and Absorption Coefficient

**Figure 3:**
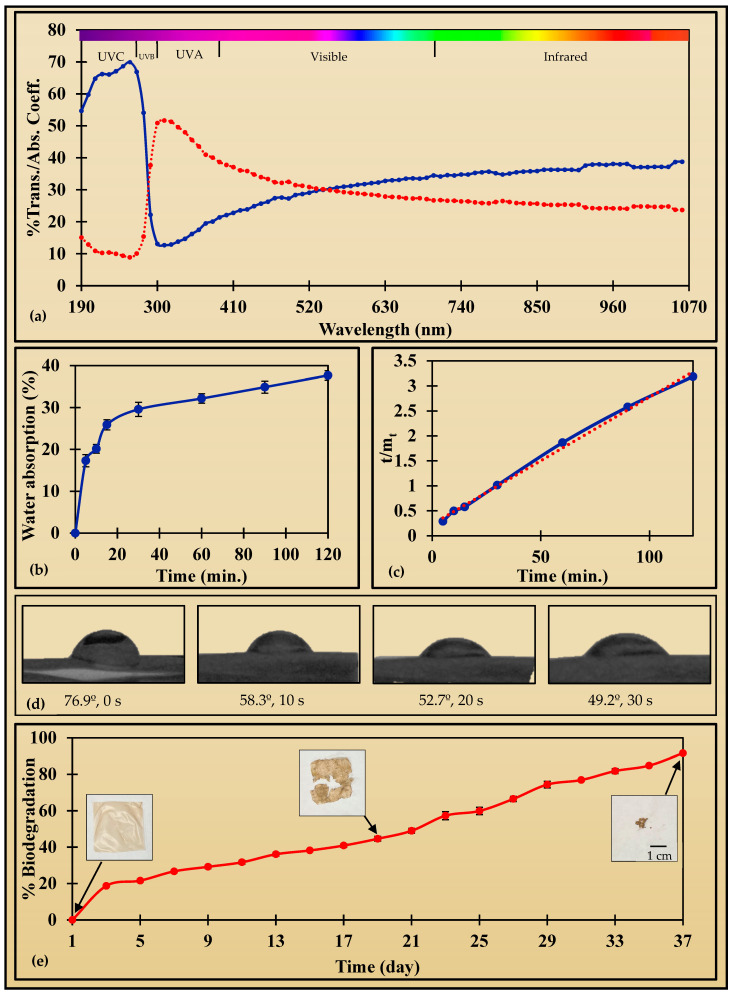
The figure displays different characteristics of the film: (**a**) %Transmittance (%Trans.) of the UV-Vis-IR radiation (solid blue line) and absorbance coefficient (Abs. Coeff.) (dotted red line), (**b**) %water absorption, (**c**) Peleg model for water absorption kinetics, (**d**) water contact angle with time 0, 10, 20, and 30 s, and (**e**) %biodegradation as a function of time, with an overlaid film images of 1st day (0% degradation), 21st day (49%), and 37th day (91.6%). The scale on the 37th day image also corresponds to the other two pictures.

The optimized soyhulls extract film was measured for transmittance at UVC, UVB, UVA, visible, and IR regions with wavelengths ranging from 190 to 1000 nm (Figure 3a). The film reduces light transmission in all these regions, with selective transmittance across different regions. It was 54.7 ± 1.3% at 190 nm, increasing to 66.9 ± 0.9% at 270 nm, then significantly dropping to 12.7 ± 1.2% at 310 nm. Then, it slightly increased to 17.5 ± 1.6% at 360 nm, followed by further increases to 30 ± 1.5% at 540 nm, 34.8 ± 1.9% at 740 nm, and reaching 37.1 ± 1.7% at 1000 nm. This selective blocking suggests that the film can protect from harmful UV rays while allowing some transparency in the visible and IR regions. These characteristics make the soyhull extract film a potentially valuable material for sustainable packaging that balances UV protection with visibility. The transparency of the SHE-optimized film was 37.6 ± 0.6% mm^−1^. This value is significantly lower than that of avocado peel fiber films with a transparency of 86.7–89.2% mm^−1^ [43] and compares well to banana peel fiber films with a transparency of 31.9–45.1% mm^−1^ [44].

Furthermore, the optimized film’s absorption coefficient was evaluated across all the regions: UVC, UVB, UVA, visible, and IR (Figure 3a). In the UVC region at 190 nm, the absorption coefficient was 15.1 ± 0.6, which increased to 50.9 ± 2.4 at 300 nm in the UVB region, 43.6 ± 2.2 at 360 nm in the UVA region, further decreased to 27.4 ± 1.2 at 680 nm in the visible region, and reached 24.1 ± 0.2 at 680 nm in the IR region. These values are notably higher than the cellulose/lignin films with an absorption coefficient between 0.4 and 9.1 at 280 nm [50].

#### 3.2.4. Antioxidant Property

Antioxidants can prolong the shelf life of food products by minimizing their oxidation. They act by inhibiting or neutralizing free radicals, which are responsible for triggering the degradation processes in food [64]. The radical scavenging activity of soyhull-extracted optimized film was determined to be 4.69 ± 0.29% and IC50 0.41 ± 0.05 g/mL. Several other studies have reported similar IC50 values of 0.037 g/mL in corncob cellulosic residue film [38], 0.11 g/L in soyhulls cellulosic residue film [35], and 0.042–0.082 g/L in spent coffee grounds films [42]. However, to enhance the antioxidant properties in developing active films, generally, the fruit/berries/green tea extracts are added to biopolymers. The IC50 values of films were higher, such as 0.09–0.31 mg/mL in corn starch/cellulose acetate with tea extract [65], 0.28 mg/mL in tamarind seed starch with red grape pomace extract [66], 0.25 mg/mL in polycarolactone/polylactic acid with green tea extract [67], 0.04 mg/mL in cellulose acetate with garlic essential oil [68], and 1.44 mg/mL in cellulose/gelatin/matcha with clitoria flower [69].

#### 3.2.5. Moisture Content and Solubility

Moisture content measurement in biodegradable films is necessary as it affects mechanical and barrier properties. Higher moisture reduces durability and increases permeability, which can shorten the shelf life of food products. It may also alter transparency and accelerate biodegradation. The moisture content of the SHE-optimized film was 12.9 ± 1%. This value of moisture is higher than that of cellulose/chitosan/curcumin films of 0.25–0.32% [70], comparable to that of corncob cellulose films of 10.1% [38] and gelatin/sodium alginate/chitosan films of 12.77–14.74% [71], and lower than that of avocado peel fiber films of 14.15–20.82% [43].

Water solubility (WS) is the film’s tendency to dissolve in water. Films with high WS dissolve or disintegrate upon contact with water, which is desirable in applications like edible films. However, low WS is preferable for applications requiring moisture resistance. The WS of optimized soyhull extract film was 26 ± 0.3%, substantially higher than that of 0.13% of cellulose/chitosan/curcumin films [70]. The reported value is significantly lower than 25.3–89.9% of starch/polyvinyl alcohol films [72]. Similarly, the WS of hake protein films and banana peel fiber films was reported to be 43.4% [73] and 24.45–65.71% [44], respectively. Relatively, the lower value of soyhull extract film may be attributed to the higher crosslinking of Zn-cellulosic chains by calcium ions, leading to a strong cellulosic network that resists the easy transport of water molecules [37].

#### 3.2.6. Water Absorption

The optimized film of the soyhull extract film was tested for water absorption (WA) properties. The WA was 17.3 ± 1.4% at 5 min, which slowly increased to 20.1 ± 1, 25.9 ± 1.2, 32.2 ± 1.1, and 34.9 ± 1.4% at 10, 15, 30, 60, and 90 min, respectively, and finally reached 37.7 ± 1.1% at 120 min (Figure 3b). Due to the presence of hydroxyl functional groups, cellulose polymers readily interact with water [35]. The maximum water absorption capacity of brown rice starch/chitosan biodegradable film was reported to be 162.92% at 8 min [74]. Furthermore, the kinetics of water absorption were studied using nine different models. The R^2^ and RMSE values of all the models are given in Table 5. The Pilosof, Gornicki, Peppa, and Czel and Czigany models showed good fitting but with an R^2^ value of around 0.90. However, the Peleg model best explains water absorption by the soyhull extract film with R^2^ and RMSE values of 0.9917 and 0.0665, respectively (Figure 3c). Similarly, the WA of films from biopolymers such as corncob cellulosic residue [38], microcrystalline cellulose [37], cellulose/ulvan [75], soyhulls cellulose [35], and spent coffee grounds [42] also follow the Peleg model.

#### 3.2.7. Water Contact Angle

The contact angle is the angle made by the liquid surface and the point of intersection between the liquid and solid surface. It is the quantitative measurement of the wetting of the solid surface using liquid. The contact angle measurement gives the surface properties of the solid surface. In particular, surface hydrophilicity or hydrophobicity is measured. The WCA of the optimized SHE film was evaluated at intervals of 0, 10, 20, and 30 s to observe its behavior over time. A WCA below 90° suggests a hydrophilic surface and more than 90° means hydrophobic. Initially, the WCA of SHE films was 76.9 ± 1.8°, which decreases to 58.3 ± 1.3° at 10 s, 52.7 ± 1° at 20 s, and 49.2 ± 1.3° at 30 s (Figure 3d). This gradual decline in WCA over time reveals an increase in the film’s hydrophilicity. Starch films have also exhibited a comparable decrease in WCA over time [45]. WCAs of 46.2–81.3° [50], 72.6° [35], 67.9–86.3° [76], 53.3–82.7° [77], and 63.4° [38] were reported in cellulose/lignin, soyhulls cellulose, chitosan, litchi seed starch–tamarind kernel, and corncob cellulose films, respectively. However, the surface of low-density polyethylene is hydrophobic with a WCA of 100.7° [78]. The hydrophobicity of SHE-optimized film can be improved by incorporating hydrophobic materials such as lignin and waxes, but further research is necessary to establish the concept.

#### 3.2.8. Biodegradation

Biodegradability is an essential measure to assess the environmental friendliness of packaging materials. The optimized SHE film biodegraded at 24% soil moisture with a weight reduction of 91.6 ± 0.9% on the 37th day. The weight loss was 18.7 ± 0.3% on the 3rd day, which steadily increased to 26.7 ± 0.7, 31.7 ± 1, 38.2 ± 0.1, 44.6 ± 1.3, 57.3 ± 2.2, 66.5 ± 1.4, 76.9 ± 0.6, 84.8 ± 0.5% on the 7th, 11th, 15th, 19th, 23rd, 27th, 31st, and 35th day, respectively (Figure 3e). Similar 91.66–96.41% biodegradation results were reported in carboxymethyl cellulose packaging films [79], and cornstarch/gelatin films biodegraded completely in 3–5 weeks [80].

Furthermore, biodegradation kinetics were analyzed using the first- and second-order models. The R^2^ and RMSE values for the first-order model were 0.9846 and 0.0589, respectively, while for the second-order model, they were 0.9949 and 0.0338 (Table 5). Thus, the second-order model more accurately explains the biodegradation kinetics of the SHE-optimized film. Likewise, the biodegradation of films from corncob cellulosic residue [38], microcrystalline cellulose [37], soyhulls cellulosic residue [35], and switchgrass lignocellulosic residue [56] follow the second-order model. In addition, using the second-order model, the half-life of the SHE-optimized film was determined to be 21.6 days.

## 4. Conclusions

This study explores the valorization of agri-industrial byproduct soyhulls to develop biodegradable packaging films. The results showed that the increased concentration of CaCl_2_ and glycerol improved the mechanical and barrier properties of films. The film was translucent, flexible, and hydrophilic, it blocked UVB light, and followed the Peleg model kinetics of water absorption. It biodegraded in 37 days at 24% soil moisture. Overall, the outcome offers the design and development of novel plastic replacement films using agri-industrial byproducts, promoting sustainability and the circular economy.

## Figures and Tables

**Table 1:** The film sample run with the combination based on independent variables: soyhull extract (SHE) in g, CaCl_2_ in mM, and glycerol in % given in terms of coded numbers for A, B, and C, respectively, and actual quantity that resembles the coded number; with response to tensile strength (TS) in MPa, elongation at break (EB) in %, and water vapor permeability (WVP) in 10^−11^ g·m^−1^·s^−1^·Pa^−1^.

Sample Run	Independent Variables						Responses		
	Coded Number			Actual Quantity					
	A	B	C	SHE	CaCl_2_	Glycerol	TS	EB	WVP
SHE1	−1	−1	0	0.3	200	1	4.1 ± 0.7	4.2 ± 0.4	4.4 ± 0.1
SHE2	1	−1	0	0.5	200	1	7.6 ± 1.2	7.1 ± 0.9	3.9 ± 0.5
SHE3	−1	1	0	0.3	500	1	9.4 ± 2.9	3.3 ± 1.5	3.4 ± 0.1
SHE4	1	1	0	0.5	500	1	10.4 ± 0.1	5.7 ± 0.7	3.3 ± 0.2
SHE5	−1	0	−1	0.3	350	0.5	8.4 ± 1.4	7.3 ± 1.2	4.0 ± 0.1
SHE6	1	0	−1	0.5	350	0.5	9.2 ± 1.1	8.2 ± 0.7	3.8 ± 0.3
SHE7	−1	0	1	0.3	350	1.5	7.5 ± 2.7	6.1 ± 0.2	4.2 ± 0.1
SHE8	1	0	1	0.5	350	1.5	8.4 ± 0.5	6.8 ± 0.5	4.0 ± 0.1
SHE9	0	−1	−1	0.4	200	0.5	5.6 ± 1.3	9.2 ± 0.7	3.9 ± 0.2
SHE10	0	1	−1	0.4	500	0.5	10.1 ± 0.2	5.1 ± 1.5	3.3 ± 0.6
SHE11	0	−1	1	0.4	200	1.5	6.2 ± 1.2	10.8 ± 0.8	3.7 ± 0.1
SHE12	0	1	1	0.4	500	1.5	12.3 ± 0.1	6.4 ± 1.0	3.4 ± 0.1
SHE13	0	0	0	0.4	350	1	6.0 ± 1.8	13.1 ± 0.7	4.0 ± 0.7
SHE14	0	0	0	0.4	350	1	7.0 ± 0.5	12.2 ± 0.3	3.8 ± 1.0
SHE15	0	0	0	0.4	350	1	6.6 ± 0.3	13.0 ± 0.8	3.7 ± 0.2

**Table 2:** ANOVA table for model fitting.

Response	Source	F-Value	*p*-Value	R^2^
TS	Model	6.86	0.0236	0.9251
	Lack of fit	5.70	0.1529	
EB	Model	6.44	0.0270	0.9206
	Lack of fit	13.57	0.0694	
WVP	Model	5.51	0.0374	0.9084
	Lack of fit	1.39	0.4448	

**Table 3:** Comparison of the tensile strength (TS, MPa), elongation at break (EB, %), and water vapor permeability (WVP, 10^−11^ g·m^−1^·s^−1^·Pa^−1^) of some cellulose-based films. The values are comparable to soyhull films, making soyhulls an important lignocellulose resource for developing biodegradable packaging films.

Biopolymer	TS	EB	WVP	Reference
Avocado peel cellulose fiber	7.2–15.7	5.2–13.6	2.4–2.5	[43]
Banana peel cellulose fiber	16.3–31.3	4.9–13.0	2.4–3.5 × 10^4^	[44]
Carboxylated cellulose nanofiber	114.2	6.5	-	[45]
Carboxymethyl cellulose (CMC)	5–19	65–82	8.8–19.4 × 10^4^	[46]
Cellulose nanofiber	7.6–15.2	13.6–27.7	1–7	[47]
Cellulose–chitosan	7.5–11.1	81.4–173.9	1.5–1.8	[48]
Cellulose–curcumin	56.0–59.5	10.5–18.8	7.3–8.4	[49]
Cellulose–lignin	25.1–110.4	2.5–8.0	11–21	[50]
CMC-PVOH	1.4–6.4	25.5–93.2	30.1–58.3	[51]
CMC–starch	6.5–20.8	25.9–37.6	11.1–99.9	[52]
Corncob cellulose	1.1–6.8	10.4–18.0	17–23	[38]
Kombucha bacterial cellulose	50–100	0.4–2.2	-	[53]
Microcrystalline cellulose	0.3–22.4	4.3–13.2	6–122	[37]
Seaweed cellulose	23.1–123.0	17.5–19.4	-	[54]
Soyhull cellulose	0.7–6.3	15.7–31.7	7–19	[35]
Soyhull lignocellulose	4.1–12.3	4.2–13.1	3.3–4.4	This work
Spent coffee ground lignocellulose	8.4–26.8	3.8–7.9	8–18	[42]
Switchgrass cellulose	8.9–12.7	2.2–2.4	16–26	[55]
Switchgrass lignocellulose	9.9–14.7	3.4–4.7	1.4–2.2	[56]
Wheat straw cellulose	5.3–6.6	16.4–27.3	19–24	[57]

**Table 4:** Film’s color values in Hunter scale.

Hunter Color Scale	Value
L*	77.2 ± 1.9
a*	4.7 ± 1.3
b*	10.7 ± 0.6
Whiteness index	74.4 ± 1.9
Yellowness index	19.9 ± 0.9
Total color difference	17.3 ± 1.6

**Table 5:** Water absorption and biodegradation kinetic model fittings for the optimized film with constants (a, b, and c), correlation coefficient (R^2^), and root mean square error (RMSE).

Model	a	b	c	R^2^	RMSE
Water absorption					
Peleg (a as K_1_, and b as K_2_)	0.2303	0.0254	-	0.9917	0.0665
Singh	0.1055	0.0171	0.0578	0.9771	0.0106
Gornicki	0.1047	0.2969	0.1965	0.9771	0.0106
Pilosof	0.1047	0.2969	17.1417	0.9771	0.0106
Czel and Czigany (b as m)	0.1234	0.2375	-	0.9625	0.0138
Peppas (a as K_1_, and b as K_2_)	−0.0128	0.2208	-	0.9442	0.1989
Vega-Galvez	0.8809	1.1377	-	0	0.0697
Garcia-Pascual	10^40^	0.013	-	−1.6889	0.1182
Weibull (a as α, and b as β)	5.37 × 10^7^	6.8172	-	-	-
Biodegradation					
First-order (a as m)	0.0454	-	2.9300	0.9846	0.0589
Second order	2.7828	0.0656	−0.0005	0.9949	0.0338

## Data Availability

The data presented in this study are available on request from the corresponding author. The data are not publicly available due to privacy restrictions.
